# Dose-response relationship between carotenoid intake and risk of depressive symptoms in postmenopausal women

**DOI:** 10.3389/fpsyt.2025.1525631

**Published:** 2025-02-19

**Authors:** Xuexue Li, Su Wang

**Affiliations:** University of Harbin Sport, Harbin, China

**Keywords:** menopausal women, depression, carotenoids, horizontal research, logical regression

## Abstract

**Background:**

It is well known that postmenopausal women have an increased risk of depression, and there is a dose correlation between carotenoid intake and depression. However, there is no clear study on the relationship between carotenoids and the risk of depression in postmenopausal women. To evaluate the relationship between the prevalence of depression and carotenoid intake in postmenopausal women.

**Methods:**

The study was based on the National Health and Nutrition Examination Survey (NHANES) from 2013 to 2018 and included 1089 postmenopausal female participants. A logistic regression model was used to verify the relationship between carotenoid intake and the prevalence of depression in postmenopausal women. A restricted cubic spline (RCS) model was used to study the dose-response relationship between carotenoid intake and depression.

**Results:**

After adjusting for confounding variables, Odds Ratios (95% confidence intervals) were found for depression in the highest quartile compared with the lower quartile. Among them, the results of lutein zeaxanthin and β-cryptoxanthin were not statistically significant (P > 0.05). Total lycopene 0.29 (0.10,0.87), β-carotene 0.41 (0.18,0.94), and total carotenoid 0.25 (0.09,0.67) were negatively correlated with the risk of depression in postmenopausal women. When α-carotene intake exceeded 2.90 mg/day, it was negatively and non-linearly associated with the prevalence of depression in postmenopausal women (P-nonlinear < 0.0022). When β-carotene intake exceeded 1.06 mg/day, it was negatively correlated with the prevalence of depression in postmenopausal women. It had an L-type nonlinear relationship with the prevalence of depression (P-nonlinear < 0.0016). Total lycopene was linearly correlated with the prevalence of depression in postmenopausal women (P-nonlinear = 0.3). When the intake exceeded 2.05 mg/day, it was negatively correlated with the prevalence.

**Conclusion:**

The study found that dietary intake of sufficient α-carotene, β-carotene, lycopene, total lutein, and zeaxanthin was negatively correlated with the prevalence of depression in postmenopausal women. Still, there was no dose correlation between β-cryptoxanthin.

## 1 Introduction

Depression is an emotional disorder and one of the leading causes of global disability (1). It is estimated that the prevalence of women is significantly higher than that of men. Women 's depression experiences two peaks: one in the postpartum period and the other in the menopause (2). As the female reproductive system ages after menopause, hormonal changes cause emotional pain, sadness, and anxiety, the emotional problems of postmenopausal women are not limited to short-term discomfort, but may also affect mental health in the long term (3). Targeted research on postmenopausal women is particularly important because epidemiological estimates suggest 1.2 billion postmenopausal women worldwide by 2030 (4).

At present, the pathophysiological mechanism of depression has not been fully elucidated, and there is still a lack of reliable biological diagnostic indicators in clinical practice, which also leads to the failure of timely diagnosis and effective intervention of depression (5). Drug therapy has the risk of poor compliance, changing cardiac metabolism, and increasing body weight (6).

In recent years, more and more studies have begun to pay attention to the role of dietary supplements in the prevention and treatment of depression. Carotenoids are plant nutrients found in food, and their unique antioxidant and anti-inflammatory properties are associated with reduced risk of various chronic diseases (7). However, due to the differences in the study population, the effects of carotenoids as an antidepressant dietary supplement on depression are different (8). Some epidemiological studies have also reported the association between carotenoid intake and the risk of depression. Total carotenoid intake may be negatively correlated with the risk of depressive symptoms in American adults. Another cohort study showed that higher levels of plasma zeaxanthin, lutein + zeaxanthin, and β-carotene combinations were associated with a decreased risk of depression in older people over time (9). However, these studies were only conducted for specific populations, and the results were inconsistent.

So far, there is no relevant survey on the relationship between carotenoids and depression in postmenopausal women. This data is nationally representative from the National Health and Nutrition Examination. It aims to quantify the association between carotenoids and depression in postmenopausal women and to compensate for the adverse effects of antidepressants in postmenopausal women through daily supplementation of carotenoids, as well as to provide dietary supplements for patients with potential depression.

## 2 Article types

### 2.1 Data and study participants

**Figure 1:**
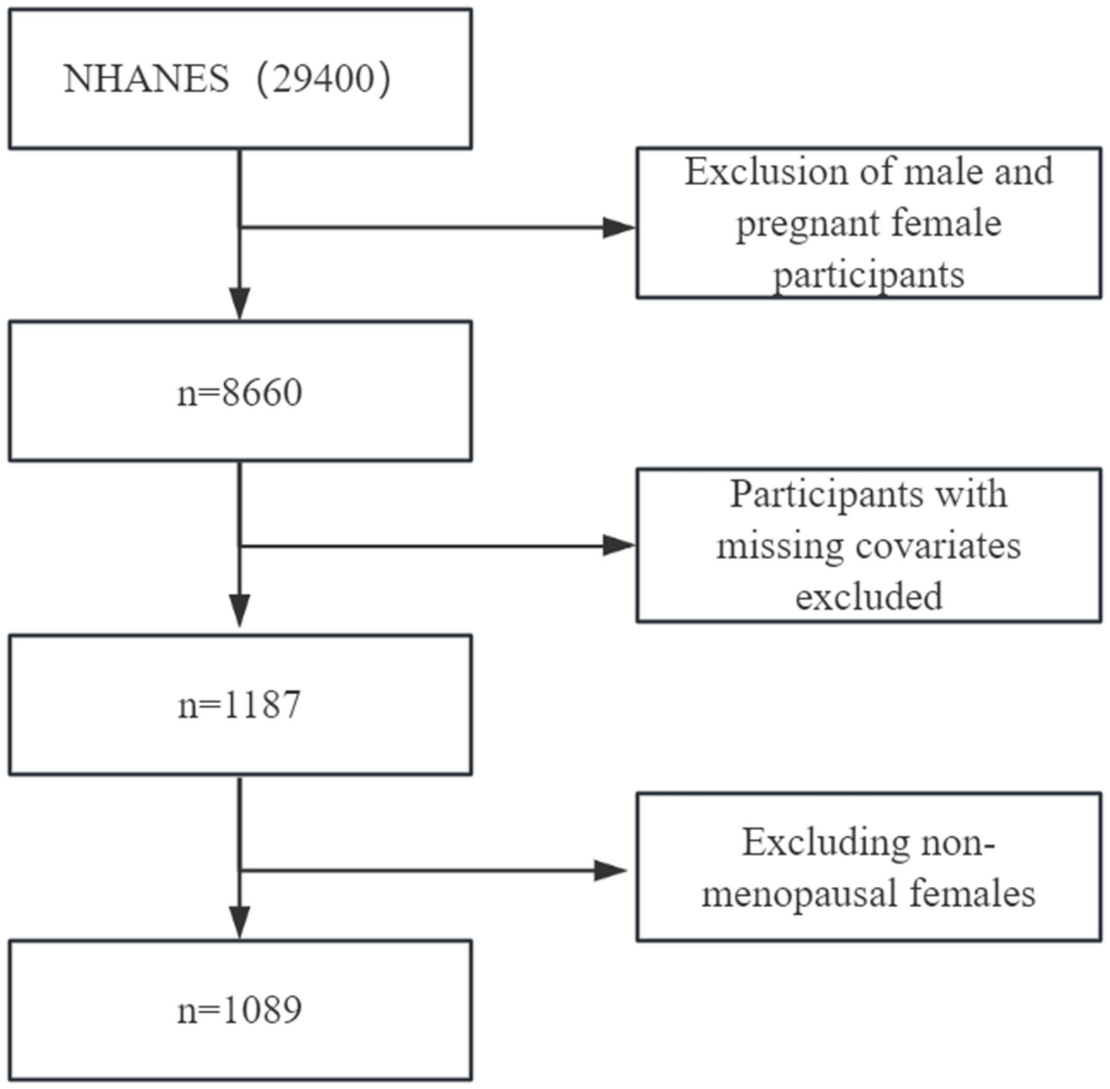
Study flowchart. Flowchart showing the process of participant selection.

The National Health and Nutrition Examination Survey (NHANES) database provided data for this survey. 29,400 participants were selected from three data cycles (2013/2014, 2015/2016, and 2017/2018). Pregnant participants under the age of 20 and females were excluded (pregnancy data only included female participants in the 20 - 44 year age group) and males; the remaining 8660 participants were excluded. After first excluding participants lacking education, race, drinking habits, smoking behavior, depression, physical activity level, carotenoid data, and participants who were not menopausal women, 1089 participants remained. Each participant signed a written informed consent form and indicated their willingness to participate in the study. Each participant signed a written informed consent form and indicated their desire to participate in this study. The specific screening process is shown in **Figure 1** below.

### 2.2 Evaluation of menopausal women

Menopause is classified as secondary, which is brought on by medical reasons, and natural, which occurs spontaneously when middle-aged women's ovarian function completely stops. Early menopausal syndrome development in women can also result from early menopause (10). The Reproductive Health Questionnaire questions whether no menstrual period has occurred in the last 12 months (excluding bleeding from surgery and medication) and whether the absence of menstruation in the last 12 months was caused by menopause/life changes or hysterectomy; only subjects who met both of these conditions were considered postmenopausal.

### 2.3 Depression score

The Patient Health Questionnaire (PHQ-9) was used to measure depression scores to determine the subjects' level of depression; only subjects who met all of these requirements were classified as menopausal women. The PHQ-9 questionnaire consists of nine questions designed to determine the frequency of depressive symptoms in the past two weeks, with each question corresponding to a score of 1-3 on a scale of 0-27. If the total PHQ-9 score is greater than or equal to 10, the subject is diagnosed with depressive symptoms (11). The effectiveness and reliability of PHQ-9 in depression screening have been validated, and it is regarded as an indispensable screening tool in primary care services in many countries. It has played an essential role in horizontal screening and provided long-term effectiveness in longitudinal studies (12).

### 2.4 Carotenoid

Carotenoids were derived from the dietary interview component of the study, and data were collected through two 24-hour dietary recall interviews. The first interview was in person in a mobile examination van, and the second was over the phone over three to ten days. To guarantee that the subjects honestly described their intake, the primary components of the study involved inquiring about the types and amounts of food ingested by the subjects and offering a range of utensils in the MEC. We utilized guidelines supplied by the USDA to convert carotenoids found in foods and drinks. This study studied the use of dietary supplements, including lutein with zeaxanthin and Lycopene, as well as the intake of alpha-carotene, beta-carotene, beta-cryptoxanthin, Lycopene, and total carotenoid in daily meals. The daily intake was determined by taking the mean of the two interviews. If the second data set was unavailable, the first interview was utilized. All numerical units are unified in milligrams (mg) for easy observation.

### 2.5 Covariates

Chronic Cardiovascular Disease (CVD): Heart failure, coronary heart disease, angina, and heart attacks were among the conditions that participants self-reported having experienced. Subjects with diabetes mellitus self-reported having received a medical diagnosis for the condition in the past. The individual self-reported having been informed by a physician or other healthcare provider that their blood pressure was high. Subjects who had suffered a stroke episode indicated as much in their self-report. For the covariates above, missing values were not eliminated to maintain the sample size; instead, missing values were interpreted as patients not afflicted with the relevant disease. Additional covariates took the form of categorical variables containing sociodemographic data: age (30–50 years, 50–70 years, 70–80 years), education level (less than 9th grade, 9th–11th grade, high school graduate/GED, some college or A.A. degree or college graduate or above), ethnicity (Mexican American, Other Hispanic, Non-Hispanic White, Non-Hispanic Black, or Other/multiracial), and lifestyle choices (none, 1–5 drinks/month, 5–10 drinks/month, or 10+ drinks/month), and BMI (underweight (<18.5 kg/m^2^), average (18.5 to <25 kg/m^2^), overweight (25–30 kg/m^2^), or obese (30 kg/m^2^ or greater). Only BMI was determined by MEC measurements from the covariates gathered through questionnaires.

### 2.6 Statistical methods

Initially, descriptive statistics—which express categorical variables as means and percentages—provided comprehensive statistics on the subjects' primary data. The percentages were presented in an unweighted format. On the other hand, means and standard deviations (S.D.s) were used to characterize continuous variables. The relationship between carotenoids and menopausal women's prevalence of depression was examined using binary logistic regression models. Two models were used to adjust all analyses. Model 2 was modified for characteristics such as diabetes, hypertension, stroke, cardiovascular disease (CVD), age, education level, ethnicity, smoking and drinking habits, and BMI, while Model 1 was not. In Model 2, we evaluated the association between the carotenoid dose and the incidence of depression in women going through menopause using restricted cubic spline statistics. All test findings were deemed significant when the two-tailed p-value was less than 0.05. R was the statistical program of choice for all studies.

## 3 Results

**Table 1** uses descriptive statistics to present the fundamental data about the participants; only 1089 menopausal women were included in the study from 2013 to 2018 based on the inclusion-exclusion criteria. Merely 13% of postmenopausal women had depression, with the majority of cases occurring in those between the ages of 50 and 70, accounting for 70% of all depressed patients. Perhaps due to the large number of non-Hispanic white participants in the study (74%), the percentage of non-Hispanic whites was 67%. Of the total, 43% of menopausal women were fat, and 65% of those who had depression also had obesity. Regarding alcohol use indications, 48% of menopausal women reported consuming one to five drinks every month; over half of them did not smoke, while 44% of the patients with depression were frequent smokers. In terms of education, tertiary-level qualifications made up 37% of the overall population and almost 40% of individuals with depression. More significant proportions of those who engaged in physical activity for less than 300 minutes per week and more of those who engaged in physical activity for less than 150 minutes per week than those who did not experience depression were linked to physical activity of over 300 minutes per week as well as a lower risk of depression. Sixty-two percent of menopausal women and around half of those who experience depression also have high blood pressure. The PHQ-9 questionnaire findings showed a mean score of 14.282, which aligns with the mild depression criteria (PHQ-9 score 10–14). In postmenopausal women, the daily carotenoid intake of depressed patients was significantly lower than that of non-depressed patients, and the daily carotenoid intake of depressed patients was about half of that of non-depressed patients. Specifically, postmenopausal women without depression consumed an average of 10 mg of total carotenoids, 4.69 mg of lycopene, 2.183 mg of lutein and zeaxanthin, 0.499 mg of α-carotene, 2.593 mg of β-carotene, and 0.107 mg of β-cryptoxanthin per day. In contrast, the daily intake of β-cryptoxanthin in patients with depression was low, only 0.07 mg/day.

**Table 1:** Characteristics of NHANES participants, 2013-2018.

Characteristic	0, N = 943 (87%)^1^	1, N = 146 (13%)^1^	p-value^2^
Age.group			0.086
30-50 years	97 (10%)	21 (16%)	
50-70 years	611 (67%)	103 (70%)	
70-80 years	235 (23%)	22 (14%)	
Race			0.083
Mexican American	111 (4.9%)	21 (5.0%)	
Other Hispanic	116 (4.1%)	28 (7.5%)	
Non-Hispanic White	419 (75%)	62 (67%)	
Non-Hispanic Black	191 (9.3%)	20 (6.2%)	
Other/multiracial	106 (7.0%)	15 (14%)	
BMI.group			0.002
Underweight(<18.5 kg/m^2^)	11 (1.3%)	1 (0.2%)	
Normal(18.5 to <25 kg/m^2^)	239 (31%)	24 (19%)	
Overweight(25 to <30 kg/m^2^)	270 (28%)	26 (16%)	
Obese(30 or greater kg/m^2^)	423 (40%)	95 (65%)	
Alq.group			0.615
1-5 drinks/month	407 (47%)	77 (57%)	
10+ drinks/month	106 (17%)	13 (15%)	
5-10 drinks/month	51 (6.1%)	5 (5.1%)	
Non-drinker	379 (30%)	51 (23%)	
Smoke.group			<0.001
Current smoker	145 (14%)	50 (44%)	
Former smoker	238 (30%)	38 (25%)	
Never smoker	560 (56%)	58 (31%)	
Education.attainment			0.099
Less Than 9th Grade	70 (3.7%)	19 (4.5%)	
9-11th Grade	105 (7.7%)	21 (11%)	
High School Grad/GED	216 (23%)	36 (29%)	
Some College or AA degree	324 (36%)	53 (40%)	
College Graduate or above	228 (29%)	17 (15%)	
PA.group			0.079
<150	280 (28%)	52 (32%)	
150-300	159 (15%)	26 (23%)	
300+	504 (57%)	68 (45%)	
Diabetes	164 (12%)	36 (26%)	0.002
CVD	79 (7.5%)	32 (24%)	<0.001
Stroke	44 (4.2%)	10 (5.0%)	0.713
Hypertension	499 (47%)	90 (62%)	0.015
PHQ	3.286 (2.271)	14.282 (3.595)	<0.001
Total	10.033 (10.552)	5.733 (5.928)	<0.001
Dietary.C	4.607 (6.529)	2.621 (3.938)	0.016
Total.C	4.649 (6.536)	2.642 (3.945)	0.014
Dietary.LZ	1.939 (3.366)	1.161 (1.902)	0.005
Supplement.LZ	1.497 (2.930)	1.050 (1.771)	0.656
Total.LZ	2.183 (3.602)	1.293 (1.997)	0.006
Dietary.A	0.499 (1.098)	0.265 (0.447)	0.003
Dietary.B	2.593 (4.510)	1.462 (2.817)	<0.001
Dietary.Y	0.107 (0.310)	0.070 (0.358)	0.005

^1^n (unweighted) (%); Mean (SD).

^2^chi-squared test with Rao & Scott's second-order correction; Wilcoxon rank-sum test for complex survey samples.

**Table 2** uses a weighted logistic regression model to show the relationship between carotenoids and depression in postmenopausal women. The variables in model 1 did not adjust the odds ratio and 95% confidence interval (CI) between the highest and lowest quartile of depression. α-carotene 0.45 (0.22,0.93) P = 0.031, β-carotene 0.30 (0.14,0.63) P = 0.002, β-cryptoxanthin 0.35 (0.16,0.76) P = 0.009, total lycopene 0.37 (0.18,0.78) P = 0.010, total lutein and zeaxanthin 0.26 (0.10,0.71) P = 0.009, total carotenoid 0.22 (0.10,0.46) P < 0.001. It is suggested that the prevalence of depression in postmenopausal women decreases with the increase in carotenoid intake. After adjusting for variables in Model 2, OR and 95% CI, α-carotene 0.56 (0.33, 0.95) P = 0.034 β-carotene 0.41 (0.18, 0.94) P = 0.037, total lycopene 0.29 (0.10, 0.87) P = 0.030. β-cryptoxanthin 0.53 (0.20,1.39) P = 0.168 total lutein and zeaxanthin 0.44 (0.13,1.46) P = 0.154 total carotenoids 0.25 (0.09,0.67) P = 0.011, β-cryptoxanthin and total lutein and zeaxanthin results were not statistically significant, see **Table 2**.

**Table 2:** Relationship between carotenoids and the incidence of depression in postmenopausal women.

Characteristic	Model 1		Model 2	
	OR (95%CI)	p-value	OR (95%CI)	p-value
Alpha-carotene				
Q1	1.00		1.00	
Q2	0.42 (0.25, 0.71)	0.002	0.51 (0.29, 0.91)	0.734
Q3	0.28 (0.13, 0.60)	0.002	0.32 (0.13, 0.82)	0.026
Q4	0.45 (0.22, 0.93)	0.031	0.56 (0.33, 0.95)	0.034
Beta-carotene				
Q1	1.00		1.00	
Q2	0.44 (0.22, 0.86)	0.017	0.47 (0.21, 1.03)	0.056
Q3	0.37 (0.16, 0.85)	0.02	0.40 (0.19, 0.85)	0.021
Q4	0.30 (0.14, 0.63)	0.002	0.41 (0.18, 0.94)	0.037
Beta-cryptoxanthin				
Q1	1.00		1.00	
Q2	0.68 (0.32, 1.45)	0.304	0.62 (0.27, 1.41)	0.216
Q3	0.59 (0.30, 1.17)	0.127	0.71 (0.33, 1.56)	0.354
Q4	0.35 (0.16, 0.76)	0.009	0.53 (0.20, 1.39)	0.168
Total lycopene				
Q1	1.00		1.00	
Q2	0.68 (0.31, 1.52)	0.336	0.67 (0.29, 1.53)	0.295
Q3	0.87 (0.39, 1.94)	0.716	0.85 (0.35, 2.06)	0.695
Q4	0.37 (0.18, 0.78)	0.010	0.29 (0.10, 0.87)	0.030
Total lutein with zeaxanthin				
Q1	1.00		1.00	
Q2	0.62 (0.33, 1.16)	0.130	0.68 (0.31, 1.50)	0.303
Q3	0.51 (0.24, 1.07)	0.074	0.69 (0.31, 1.54)	0.321
Q4	0.26 (0.10, 0.71)	0.009	0.44 (0.13, 1.46)	0.154
Total carotenoid				
Q1	1.00		1.00	
Q2	0.57 (0.29, 1.13)	0.105	0.64 (0.32, 1.26)	0.170
Q3	0.42 (0.22, 0.80)	0.010	0.45 (0.23, 0.91)	0.029
Q4	0.22 (0.10, 0.46)	<0.001	0.25 (0.09, 0.67)	0.011

1OR, Odds Ratio; CI, Confidence Interval. Model 1 univariate analysis; Model 2 was adjusted for age, education level, smoking status, body mass index, alcohol intake, hypertension, CVD, Stroke and diabetes.

**Figure 2:**
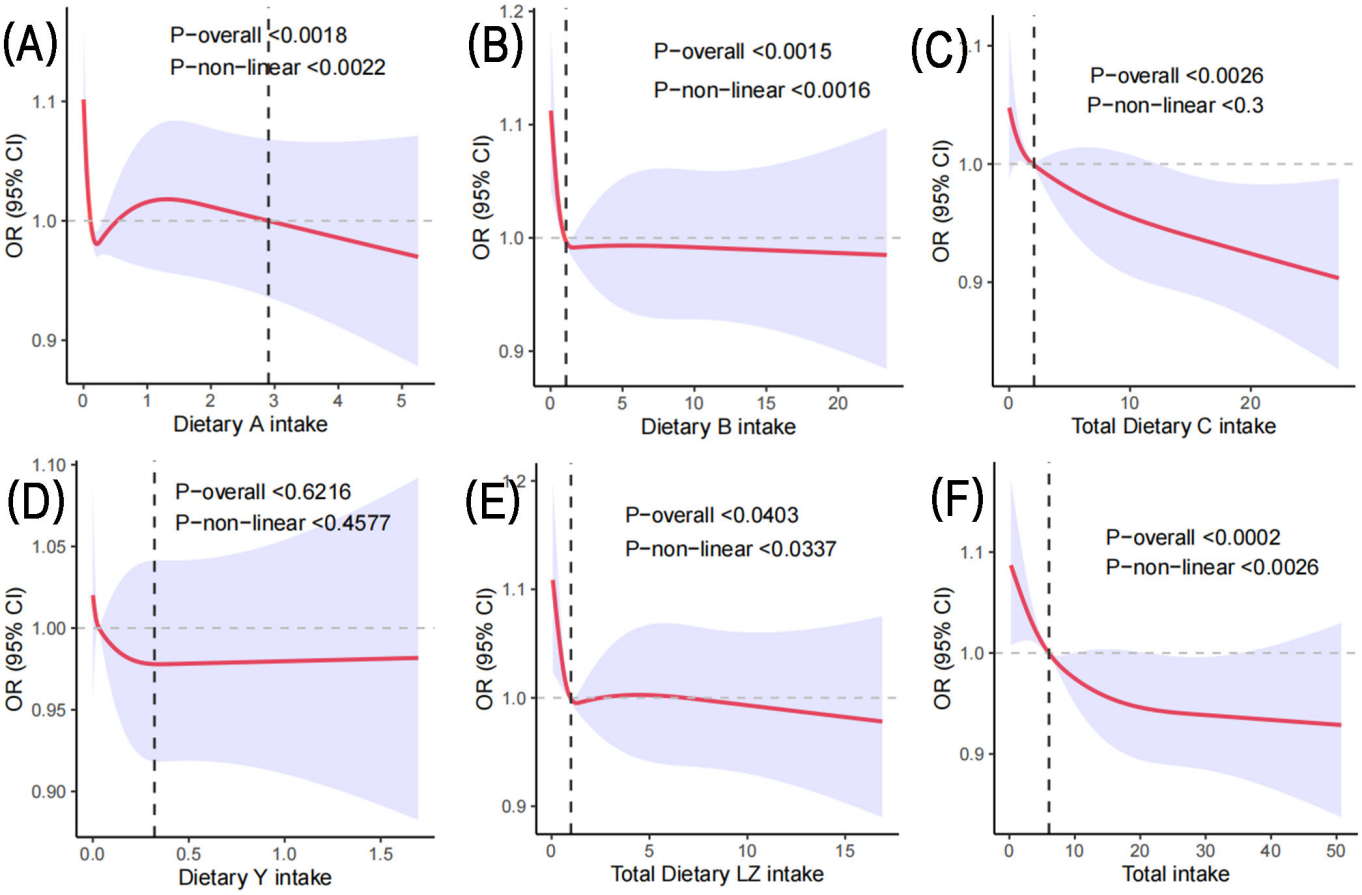
Based on Model 2, the RCS method was used to analyze the dose response of carotenoids to the risk of depression in postmenopausal women. **(A)** represents the daily intake of α-carotene; **(B)** represents the daily intake of β-carotene; **(C)** represents the daily lycopene intake; **(D)** represents the daily intake of β-cryptoxanthin; **(E)** Daily total lutein and zeaxanthin intake; **(F)** represents the daily total carotenoid intake.

**Figure 2** shows the dose-response relationship between carotenoid intake and the development of depression in postmenopausal women. Based on Model 2, the limited cubic spline statistical method was used to analyze the relationship between the intake of various carotenoids and total carotenoids in postmenopausal women and the dose of depression. In **Figure 2A**, there was a nonlinear relationship between α-carotene and the occurrence of depression in postmenopausal women (P-nonlinear < 0.0022). It can be observed that when the intake is less than 2.90 mg/day, there is a fluctuating relationship between α-carotene and the prevalence of depression in postmenopausal women. However, there was a negative correlation when the intake exceeded 2.90 mg/day. In **Figure 2B**, there was an L-shaped nonlinear relationship between β-carotene and depression (P-nonlinear < 0.0016), and the intake was greater than 1.06 mg/day, which was negatively correlated with the prevalence of depression in postmenopausal women. The total lycopene in **Figure 2C** showed a linear relationship with the prevalence of depression in postmenopausal women (P > 0.05). When daily intake was 2.05 mg, it was negatively correlated with the prevalence of depression in postmenopausal women. In **Figure 2D**, there was no significant correlation between b-cryptoxanthin intake and the prevalence of depression in postmenopausal women (P-overall > 0.05), and there was no correlation between β-cryptoxanthin intake and depression in postmenopausal women (P-nonlinear > 0.05). In **Figure 2E**, there was a nonlinear relationship between total lutein and zeaxanthin and depression in postmenopausal women (P-nonlinear < 0.0337). The correlation between the two fluctuated when the daily intake was less than 6.82 mg. More than 6.82 mg/day, it was negatively correlated. The total carotenoids in **Figure 2F** also showed a nonlinear relationship with the occurrence of depression in postmenopausal women (P-nonlinear < 0.0026), and more than 6.05 mg/day was negatively correlated with depression.

## 4 Conclusions

### 4.1 Main findings of this study

Using information from the NHANES database for three cycles from 2013 to 2018, this study found a negative correlation between the prevalence of depression and carotenoid intake in postmenopausal women. High daily intakes of α-carotene, β-carotene, total lycopene, and carotenoids were associated with a lower prevalence of depression, involving age, education level, race, smoking and drinking status, body mass index, cardiovascular disease, hypertension, stroke, and diabetes. Eating carotenoids can help postmenopausal women reduce depression. RCS analysis showed that the occurrence of depression in postmenopausal women was significantly dose-related with total carotenoids, α-carotene, β-carotene, lycopene, total lutein, and zeaxanthin. However, there was no significant dose relationship between β-cryptoxanthin intake and the prevalence of depression in postmenopausal women.

### 4.2 Impact of carotenoids on depression

There are few studies on the relationship between carotenoids and the prevalence of depression in postmenopausal women. The results of this study are similar to the relationship between depression. The results of a systematic review and meta-analysis showed that although β-cryptoxanthin was not associated with depression, total carotenoids, β-carotene, lycopene, and α-carotene did have a significant negative correlation with zeaxanthin (13). A cross-sectional study based on data from the National Women 's Health Study (SWAN) found that dietary β-carotene and α-carotene intake were negatively correlated with the prevalence of depression in middle-aged and older women (14). A cross-sectional study has shown that eating tomatoes can help Japanese elderly (≥ 70 years old) avoid depression (15). In another cross-sectional study, total carotenoids, lutein + zeaxanthin, lycopene, α-carotene, β-cryptoxanthin, and β-carotene all reduced the risk of depression in patients with cardiovascular metabolic abnormalities (16). Another study showed that β-cryptoxanthin intake was negatively correlated with depression, but there was no correlation between β-carotene, lutein, lycopene, or β-carotene plus zeaxanthin (17). In conclusion, despite the results of the study population and other confounding factors, carotenoid intake, in general, may reduce the prevalence of depression in postmenopausal women.

### 4.3 Mechanisms of carotenoids and depression

Several complex elements, including unfavourable living circumstances and family history, have a role in the pathophysiology of depression. Although it has not yet been determined precisely, oxidative stress and immuno-inflammatory pathways are commonly acknowledged as potential explanations. Because perimenopausal women experience unpredictable mental states brought on by hormonal instability, depression is more common in these women during the menopausal transition and early postmenopausal era—reference (18). As women approach menopause, they may also experience a range of symptoms of differing intensities, including joint pain, sleeplessness, night sweats, and exhaustion, these symptoms can also worsen the onset of depression (19). Research revealed a decline in the synthesis of antioxidant enzymes and increased levels of oxidative stress in postmenopausal women (20). It has also been noted that women having hysterectomy experience elevated levels of oxidative stress (21). Consequently, it is conceivable that the aging-related decrease in estrogenic causes higher amounts of oxidative stress, which increases the vulnerability of the central nervous system to damage from free radicals and raises the chance of developing depression. According to earlier research, proinflammatory factors are more prevalent in the plasma or cerebral fluid of depressed individuals than in healthy individuals. One of the critical processes behind depression is thought to be neurogenesis. Neurogenesis influences the development of resistance by affecting neurotransmission, stimulating the immune system, encouraging the growth of new neurons, managing synaptic plasticity, and regulating the synthesis of immunological components (22). Bad lifestyle choices can also result in proinflammatory elements, which can make depression worse. Experiments have shown that supplementing with extra antioxidants can successfully prevent depression and other psychiatric illnesses brought on by stress. Furthermore, poor intake of carotenoids may indicate an unbalanced dietary pattern. Carotenoids also have excellent antioxidant and anti-inflammatory capabilities. It has been shown that Lycopene plays crucial roles in illnesses of the central nervous system, among other processes being the control of oxidative stress and neuroinflammation, the restoration of mitochondrial function, and the inhibition of neuronal death (23). By raising brain-derived neurotrophic factors and lowering proinflammatory cytokine levels, β-carotene may have an antidepressant effect on the brain (24). An individual's immune function may deteriorate due to life's pressures. However, systemic or localized neuro oxidative stress can be efficiently reduced by supplementing with compounds like lutein with zeaxanthin, reducing the physiological stress response and relieving psychological stress (25). Therefore, it is clear that carotenoids play a part in depression from the antioxidant and anti-inflammatory processes. In addition, carotenoids can also affect mental health through other mechanisms (26). For example, they can promote the balance of intestinal microbial communities and maintain good digestive system functions. A healthy gut environment helps maintain a stable level of serotonin, an important ' pleasure hormone ' that plays a key role in mood regulation.

### 4.4 Other findings of this study

There was no significant correlation between the intake of total lutein and zeaxanthin and the occurrence of depression. However, through dose statistical model analysis, it was found that there was a certain dose correlation between depression and total lutein and zeaxanthin. Previous research has demonstrated that those with depression had lower lutein intakes and that the frontal lobe's total lutein and total carotenoid concentrations gradually declined with age (27). These findings may be crucial for understanding the underlying mechanisms of depression. Lutein and zeaxanthin are common carotenoids in plants, which play an important role in the prevention and treatment of neurological diseases (28). For example, lutein and zeaxanthin can resist oxidation, anti-inflammation, and protect nerve cells from free radical damage. Therefore, the above results can be partially explained that the changes of hormone levels in postmenopausal women lead to the emergence of different aging-related factors. There was no significant dose relationship between β-cryptoxanthin intake and the prevalence of depression in postmenopausal women. This conclusion is consistent with a previous review that found no significant correlation between β-cryptoxanthin intake and depressive symptoms (13). Due to the limited number of related studies, it is not yet possible to clarify the mechanism behind it. It is speculated that the likely reason is that the physiological characteristics of β-cryptoxanthin interact with its exogenous substances or endogenous molecular networks in food, thus affecting its absorption, metabolism, and bioavailability. This review also emphasizes that it is hoped that more molecular biological studies on the relationship between β-cryptoxanthin intake and depression will be needed in the future to explore the specific mechanisms further.

### 4.5 Limitations and strengths

This study uses the NHANES database to conduct a cross-sectional investigation on carotenoids and depression in menopausal women. Our study has several strengths. Firstly, the 24-hour dietary recall questionnaire used in the survey, along with multilevel sampling and weighted statistical methods, allowed participants' dietary information to be thoroughly collected. Secondly, our findings on depression in menopausal women can be applied to a national scale and are not influenced by variables such as age, education level, ethnicity, smoking status, drinking status, BMI, CVD, hypertension, stroke, and diabetes status. In the end, the study looks at a particular group of menopausal women and employs nutrition as a supplemental treatment to make up for drug deficits. However, the study still has several things that could be improved. It was not feasible to ascertain if carotenoids have a long-term effect on depression in menopausal women. Second, much of the information gathered during data collection was derived from questionnaires rather than objective measurements. Furthermore, more clinical validation is needed before any inferences can be made because this was a cross-sectional design experiment. It is worth noting that although current research results show a correlation between carotenoid intake and a lower risk of depression, this does not mean that diet alone can completely prevent or treat depression. Maintaining a balanced diet structure, combined with appropriate exercise and psychological support, is a comprehensive method to maintain good mental health.

### 4.6 Conclusion

To sum up, the research indicates that adequate dietary intake of α-carotene, β-carotene, lycopene, total lutein, and zeaxanthin was negatively correlated with the prevalence of depression in postmenopausal women. Still, there was no significant dose relationship between β-cryptoxanthin intake and the prevalence of depression in postmenopausal women. However, more clinical trials are needed to verify these findings.

## Data Availability

Publicly available datasets were analyzed in this study. This data can be found here: https://www.cdc.gov/nchs/nhanes/index.htm.
